# Observations on the Structure of the Tongue, Illustrated by Cases, in Which a Portion of That Organ Has Been Removed by Ligature

**Published:** 1804-03-01

**Authors:** Everard Home


### Observations on the Structure of the Tongue, *illustrated by Cases, in which a Portion of that Organ has been removed by Ligature. By* Everard Home, Esq. F.R.S.

PHYSIOLOGICAL inquiries have ever been considered as deserving attention; and, whenever medical practitioners, in the treatment of diseases, have met with any circumstance, which threw light upon the natural structure or actions of any of the organs of the human body, or those of other animals, their communications have met with a favourable reception.

The following observations derive their real importance from offering a safe and effectual means of removing a portion of the tongue, when that organ has taken on a diseased action, the cure of which is not within the reach of medicine; and as the tongue, like many other glandular structures, is liable to be affected by cancer, it becomes of no small importance that the fact should be generally known known. In a physiological view, they tend to show, that the internal structure of the tongue is not of that delicate and sensible nature which, from its being the organ of taste, we should be led to imagine.

The tongue is made up of fasciculi of muscular fibres, with an intermediate substance met with in no other part of the body, and a vast number of small glands; it has large nerves passing through it; and the tip possesses great sensibility, fitting it for the purpose of taste.

Whether the sense of taste is confined entirely to the point of the tongue, and the other parts are made up of muscles fitted for giving it motion; or whether the whole tongue is to be considered as the organ, and the soft matter which pervades its substance, and fills the interstices between the fasiculi of muscular fibres, is to be considered as connected with sensation, has not, I believe, been ascertained.

The tongue, throughout its substance, has always been considered by physiologists as a very delicate organ; and it was believed, that any injury committed upon it would not only produce great local irritation, but also affect, in a violent degree, the general system of the body. This was my own opinion till I met with the following case, the circumstances of which induced me to see this organ in a different point of view.

A gentleman, by an accident which it is unnecessary to describe, had his tongue bitten with great violence. The immediate effect of the injury was great local pain; but it was not attended with much swelling of the tongue itself, nor any other symptom, except that the point of the tongue entirely lost its sensibility, which deprived it of the power of taste: whatever substance the patient eat was equally insipid. This alarmed him very much, and induced him to state to me the circumstances of his case, and request my opinion. I examined the tongue a fortnight after the accident; it had the natural appearance, but the tip was completely insensible, and was like a piece of board in his mouth, rendering the act of eating a very unpleasant operation. I saw him three months afterwards, and it was still in nearly the same state.

From this case it appears, that the tongue itself is not particularly irritable; but the nerves passing through its substance to supply the tip, which forms the organ of taste, are very readily deprived of their natural action; this probably arises from their being softer in texture than nerves in general, and, in that respect, resembling those belonging to the other organs of sense. There

There was another circumstance in this case which very particularly struck my attention, viz. that a bruise upon the nerves of the tongue, sufficient to deprive them of the power of communicating sensation, was productive of no inflammation or irritation in the nervous trunk, so as to induce spasms, which too commonly occur from injuries to the nerves belonging to voluntary muscles; I am therefore led to believe, that the nerves supplying an organ of sense, are not so liable to such effects as those which belong to the other parts of the body.

The small degree of mischief which was produced, and the readiness with which the nerves had their communication completely cut off, were to me new facts, and encouraged me, in the following case of fungous excrescence from the tongue, which bled so profusely as at times to endanger the patient's life, and never allowed him to arrive at a state of tolerable health, to attempt removing the part by ligature.

John Weymouth, eight years of age, was admitted into St. George's Hospital on the 24th of December, 1800, on account of a fungous excrescence on the right side of the anterior part of the tongue, which extended nearly from the outer edge to the middle line at the tip. It appeared, from the account of his relations, that the origin of this fungus existed at his birth, and had been increasing ever since. He had been a year and a half under the care of the late Mr. Cruikshank, who had removed the excrescence by ligature round its base; but, when the ligature dropped off, a violent hæmorrhage took place, and the excrescence gradually returned. Attempts were made to destroy it by caustic; but hæmorrhagy always followed the separation of the sloughs; so that, after ten trials, this mode was found ineffectual. It was also removed by the knife, ten different times, but always returned.

From this history I was led to believe, that the only mode of removing the disease was taking out the portion of the tongue upon which it grew. This was a case in which I felt myself warranted in making an attempt out of the common line of practice, to give the patient a chance of recovery; and, from the preceding case, having found that pressure on one part of the tongue produced no bad consequences on the other parts, I was led to remove the excrescence in the following manner.

On the 28th of December, I made the boy hold out his tongue, and passed a crooked needle, armed with a double ligature, directly through its substance, immediately beyond the the excrescence. The needle was brought out below, leaving the ligatures; one of these was tied very tight before the excrescence, the other equally so beyond it, so that a segment of the tongue was confined between these two ligatures, in which the circulation was completely stopped. The tongue was thin in its substance; and the boy complained of little pain during the operation. Thirty drops of laudanum were given to him immediately after it, and he was put to bed. He fell asleep, continued to dose the greater part of the day, and was so easy the next day as to require no particular attention.

On the fifth day from the operation, the portion of tongue came away with the ligatures, leaving a sloughy surface, which was thrown off on the eleventh day, and succeeded by a similar slough; this separated on the fifteenth day. The excavation after this gradually filled up; and, on the twentieth day, it was completely cicatrized, leaving only a small fissure on that side of the tongue.

Encouraged by the result of this case, I was led to perform a similar operation upon a person at a more advanced period of life.

Margaret Dalton, 40 years of age, was admitted into St. George's hospital, on the 25th of December, 1801, on account of a tumour, the size of a pea, situated on the right side of the tongue, near its edge. The history of the case was as follows. A small pimple appeared, and gradually increased, without pain; the only inconvenience was, that it affected her speech, and, when bruised by the teeth, bled freely.

The operation was performed on the 11th of January, 1802, in exactly the same manner as has been already described. It produced a considerable degree of salivation, which was extremely troublesome (much more so than the pain the ligatures produced), and continued till the slough came away. The ligature nearest the root of the tongue separated on the sixth day; the other on the seventh; and, in three days after the separation of the second ligature, the wound was completely skinned over.

A third case of this kind came under my observation, in which there was a small tumour in the substance of the tongue, about the size of a pea, which gave me the idea of its being of that kind which might terminate in cancer. The patient was a gentleman of about forty-one years of age. Upon examining the tumour, I told him of my alarm respecting its nature; and at the same time added, that I was very ready to remove it, should it be the opinion of other practitioners that such a step was adviseable; and my experience in two former cases led me to believe it might be done with safety. I therefore advised him to consult other medical practitioners of reputation, and acquaint me with their opinion. Mr. Cline was consulted, and his opinion coincided with mine; which made the patient decide upon having the tumour removed.

The operation was performed on the 28th of December, 1802. The needle pierced the tongue an inch beyond the tip, a little to the right of the middle line of the tongue; and the space between the two ligatures, when they were tied at the circumference of the tongue, was fully an inch. The tongue was thick; and the mass included by the ligatures was such as to make it difficult to compress it. The operation gave considerable pain, of a numbing kind. Immediately after the operation, the part included became dark coloured, particularly towards the middle line of the tongue. A salivation took place. The next day, the pain and salivation were great, and the patient could not swallow; but, on the day following, he could take broth, negus, and other fluids.

On the sixth day from the operation, the slough became loose; and the least motion of the tongue gave great pain. Upon examining the slough, there was a small spot which looked red, and was surrounded by a dark surface; this was towards the right side. Upon further examination it appeared, that the ligature to the right had not completely deadened the part at the centre, in which the artery had its course. This accounted for the red spot, as well as for the pain the patient suffered; and led me, on the seventh day, to disengage the ligature on the left, (which was almost completely separated) by means of a pair of scissars, and pass another ligature through the grove to the opposite side, and tie it over the part not completely deadened. This gave great pain for a few hours, which was relieved by the use of tincture of opium. On the eighth day, the patient had less pain than on any preceding day, and less salivation; and, on the ninth, the whole slough came away. On the thirteenth, the tongue had so much recovered itself, that there did not appear any loss of substance whatever, only a fissure of half an inch in depth, in the anterior part of it; and, as that now seemed to be exactly in the centre, there was not the smallest deformity.

The preceding cases, in the view which it is intended to take in the present Paper, are to be considered as so many experiments, by which the structure of the tongue is in some respects ascertained: They enable us to draw the following conclusions.

The internal structure of the tongue is less irritable than almost any other organized part of the body; therefore, the peculiar substance which is interposed between the fasciculi of its muscular fibres, is not in any respect connected with the nerves which pass through its substance to the organ of taste, but is merely a soft medium, to admit of great facility of action in its different parts.

The nerves of the tongue appear to be more readily compressed, and deprived of their power of communicating sensation, than nerves in general; and any injury done to them is not productive of diseased action in the trunk of the injured nerve.

If we compare the effects of compression upon a portion of the tongue with those of a similar compression upon the hæmorrhoidal veins when they form piles, or those of the testicle in cases of varicose veins of the spermatic chord, which not only produce very violent local inflammation, but also a considerable degree of symptomatic fever, it is impossible not to be surprised that the results should be so very different; since we are led to believe, upon a general principle, that parts are sensible in proportion to their vascularity, and that all the organs of sense, when inflamed, are more exquisitely so than any other parts of the body.

The tongue appears to have a power of throwing off its sloughs in a shorter time than any other part. Eight or nine days is the ordinary time of a slough separating from the common parts; in the boy's tongue it was only five.

Having stated the information we derive from these cases, respecting the structure, sensibility, and irritability of the tongue, it now remains to mention the advantage to be derived from them in a professional view; and so strongly is it connected with humanity, that it cannot be undeserving the utmost attention.

The information derived from these cases, enables us to attempt with safety, the removal of any part of the tongue which may have taken on a disposition to become cancerous. As this disease in the tongue always begins in a very small portion of that organ, it is, in the early stage, more within the reach of removal than when in any other part of the body; and as the glands of the tongue are independent of each other, the cancerous disposition by which one of them is attacked, does not so readily communicate itself to the others; and the part may be removed, with a greater greater degree of security against a future recurrence of the disease, than in other cases where this malady attacks a portion of a large gland, the whole of which may be under the influence of the poison, long before there is any appearance of its being diseased.

